# Statin prescription initiation and lifestyle behaviour: a primary care cohort study

**DOI:** 10.1186/s12875-016-0471-6

**Published:** 2016-07-18

**Authors:** S. F. McAleer, M. E. Cupples, C. E. Neville, M. C. McKinley, J. V. Woodside, M. A. Tully

**Affiliations:** Centre for Public Health, Institute of Clinical Sciences Block B, Royal Victoria Hospital, Queen’s University Belfast, Grosvenor Road, Belfast, BT12 6BA UK; UKCRC Centre of Excellence for Public Health Research (NI), Institute of Clinical Sciences Block B, Royal Victoria Hospital, Grosvenor Road, Belfast, BT12 6BA UK; Department of General Practice and Primary Care, Queen’s University, Belfast, UK

**Keywords:** Cardiovascular disease, Statin prescribing, Lifestyle, Behaviour change, Diet, Physical activity, Patient education, Primary care, Cohort study

## Abstract

**Background:**

Statin prescribing and healthy lifestyles contribute to declining cardiovascular disease mortality. Recent guidelines emphasise the importance of giving lifestyle advice in association with prescribing statins but adherence to healthy lifestyle recommendations is sub-optimal. However, little is known about any change in patients’ lifestyle behaviours when starting statins or of their recall of receiving advice. This study aimed to examine patients’ diet and physical activity (PA) behaviours and their recall of lifestyle advice following initiation of statin prescribing in primary care.

**Method:**

In 12 general practices, patients with a recent initial prescription of statin therapy, were invited to participate. Those who agreed received a food diary by post, to record food consumed over 4 consecutive days and return to the researcher. We also telephoned participants to administer brief validated questionnaires to assess typical daily diet (DINE) and PA level (Godin). Using the same methods, food diaries and questionnaires were repeated 3 months later. At both times participants were asked if they had changed their behaviour or received advice about their diet or PA.

**Results:**

Of 384 invited, 122 (32 %) participated; 109 (89.3 %) completed paired datasets; 50 (45.9 %) were male; their mean age was 64 years. 53.2 % (58/109) recalled receiving lifestyle advice. Of those who did, 69.0 % (40/58) reported having changed their diet or PA, compared to 31.4 % (16/51) of those who did not recall receiving advice. Initial mean daily saturated fat intake (12.9 % (SD3.5) of total energy) was higher than recommended; mean fibre intake (13.8 g/day (SD5.5)), fruit/vegetable consumption (2.7 portions/day (SD1.3)) and PA levels (Godin score 7.1 (SD13.9)) were low. Overall, although some individuals showed evidence of behaviour change, there were no significant changes in the proportions who reported high or medium fat intake (42.2 % v 49.5 %), low fibre (51.4 % v 55.0 %), or insufficient PA (80.7 % v 83.5 %) at 3-month follow-up.

**Conclusion:**

Whilst approximately half of our cohort recalled receiving lifestyle advice associated with statin prescribing this did not translate into significant changes in diet or PA. Further research is needed to explore gaps between people’s knowledge and behaviours and determine how best to provide advice that supports behaviour change.

**Electronic supplementary material:**

The online version of this article (doi:10.1186/s12875-016-0471-6) contains supplementary material, which is available to authorized users.

## Background

Recently there has been significant professional and public controversy regarding the appropriate use of statins in cardiovascular disease prevention [1, 2]. Statin prescriptions have increased exponentially over the past decade [3] and statin prescribing is recommended at increasingly lower levels of cardiovascular risk, [4, 5] in association with the provision of appropriate lifestyle advice. Little is known, however, of the impact of lifestyle advice or of patients’ behaviour around the time of initiating statin therapy in primary care.

For both primary and secondary prevention of cardiovascular disease, American, British and European guidelines [4–6] present evidence of the value of healthy diets and of physical activity (PA). These guidelines target a collaborative multi-sectoral approach which includes primary healthcare professionals. Although there has been recent debate regarding the role of saturated fat in cardiovascular disease [7] the guidelines currently recommend that patients are advised that their total dietary fat intake should be less than 30 % of their total energy intake, [4, 5] with saturated fat comprising less than 7 % of the total fat. However, there is an increasing body of epidemiological evidence that supports advice regarding the value of dietary fibre in preventing cardiovascular disease [8]. The consumption of fresh fruit, vegetables and adherence to a Mediterranean diet have been shown to reduce mortality [9]. Also, guidelines recommend that adults should take at least 150 min per week of moderate intensity physical activity to promote and maintain their health [10, 11].

Studies have shown that much of the recent decline in cardiovascular mortality worldwide, including the UK [12, 13] where expenditure on statin prescriptions is highest of all European countries [3], is attributable to changes in risk factors, independent of prescribed medication. However, surveys indicate that patients’ adherence to diet and physical activity guidance is sub-optimal [14]. Evidence suggests that lifestyle change can enhance the effect of statin medication in lowering cholesterol levels [15] but also that the lifestyle behaviours of statin users do not differ from non-users [16]. There is therefore a need for increasing emphasis on the importance of healthy lifestyles and on the role of health professionals in providing their patients with lifestyle advice and support for behaviour change [6].

Whilst the impact of effective health promotion interventions and adherence to lifestyle advice [17, 18] tend to fall over time, a previous study found no significant change in dietary fat intake after six months of statin use in a US population [19]. However, there is a lack of evidence regarding the extent to which patients in the UK recall advice or change their lifestyle behaviours following initiation of statin therapy.

The aim of our study was to examine, in a UK primary care population, patients’ recall of lifestyle advice and their diet (fibre and fat consumption) and PA behaviours following the initiation of statin therapy.

## Method

### Participants

All 12 general practices, which we selected purposively to include a range of socio-economic and cultural settings in Northern Ireland, accepted our invitation to participate in the study. Their list sizes ranged from 4,000 to 10,000 patients. Eligible patients were identified over a 4-month period by practice staff searching electronic records, using a specified search strategy every two weeks. Inclusion criteria included patients who were fully registered in the practices, aged >18 years, prescribed statin medication in the previous 4 weeks (either for primary or secondary prevention) and with no previous statin prescription. Patients who were unable to answer a telephone questionnaire or complete a food diary, non-English speaking, living in an institution or without a telephone number, were excluded.

### Process and data collection

Participants received their usual healthcare throughout the study and did not receive any additional advice from the researcher. Practices posted study information and letters of invitation to potential participants during August to November 2005. Only one invitation was sent: ethics approval was not given for reminders, nor for the researchers to examine patients’ medical records. Those who agreed to participate returned a signed response in a freepost envelope to the researcher who posted a food diary to them and, between 72 and 96 h later, telephoned them to confirm consent, explain how a food diary was to be completed and administer validated questionnaires to assess diet (Dietary Intervention in Primary Care (DINE) [20]) and PA (Godin [21]). The researcher also asked them if they had made any change to their diet or physical activity habits during the previous 3 months and if they had received advice from hospital, GP surgery or other sources and, within these options, if it was given by a doctor, nurse or other health professional.

The DINE food frequency questionnaire assesses the frequency of consumption of 19 food groups in a common Western diet and provides scores for fat and fibre intake. Each food group is given a score to reflect the nutrient content of a standard portion size, and scores are weighted according to frequency of consumption. The Godin questionnaire provides a measure of type, duration and intensity of leisure-time exercise [21]: scores are constructed on the basis of numbers of 15-min episodes of PA of different intensities in the previous week. Scores <14 units indicate insufficient PA, with low health benefits; 14–23 indicates some PA; ≥24 indicates sufficient PA with substantial health benefits.

Those who had not returned their food diary after 14 days received a telephone reminder, repeated after a further 10 days. The first food diary and questionnaires were completed approximately 1 month after the initial statin prescription. Three months later a further 4-day food diary was posted to each participant and the DINE and Godin questionnaires were repeated, by telephone.

Based on data from the National Diet & Nutrition Survey [22] we determined that 92 paired repeat observations would allow detection of a 5 g change in saturated fat intake, approximately equivalent to a 1 % change in its contribution to total daily energy consumption, with 90 % power and an alpha of 5 %. To allow 25 % attrition, we planned to recruit 120 participants; expecting a 30 % response rate to postal invitations, we planned to invite approximately 400.

### Data management and analysis

Scores for DINE and Godin questionnaires were computed using recommended methods [20, 21]. Data from the food diaries were coded, entered and analysed using WISP software (WISP, Tinuviel Software, Warrington), converting data manually to quantities, where no specific weight was recorded, using average food portion size charts [23]: measurements were expressed as percentages of total daily energy intake and were interpreted in relation to UK reference values for nutrient intake [24]. Analyses were conducted using SPSS v 19. Paired samples t-tests were used to assess changes over time; chi squared tests were used to compare individuals’ change in categories of diet and PA scores. Intracluster correlation coefficients (ICCs), based at practice level, were calculated for changes in diet and physical activity and recall of advice. Comparisons of DINE and food diary assessments were made by Spearman’s rho analysis and Kappa.

## Results

### Characteristics of respondents

We invited 384 individuals to participate; 122 (32 %) were recruited, 3 were excluded (due to deafness, illiteracy and no telephone respectively); 259 did not respond. The number recruited from each practice varied, ranging from 1 to 20 (median 9). Of the 122 recruited, 89.3 % (109) provided paired questionnaire data; 75.4 % (92) returned paired completed food diaries, valid for analysis. All 122 completed the first questionnaires but 8 did not complete second questionnaires (7 due to concurrent morbidity; 1 could not be contacted). Two did not return a first food diary, 10 declined to complete a second but a postal strike led to inappropriate timing of completion of 3 first and 7 sec food diaries.

Participants’ ages ranged from 38 to 84 years (mean 64 years); 50 (45.9 %) were male (Table 1). There was no significant difference between the age or sex distributions of the respondent and non-respondent samples (p > 0.05): the respondent sample included a smaller proportion of the most socio-economically disadvantaged quartile of the general population (34.0 % v 43.6 %) but this difference was not of statistical significance (p > 0.05). Based on self-report 82 % of respondents had a history of high blood pressure, angina, myocardial infarction, stroke or diabetes (some had more than one of these conditions); 22 % were current smokers.

**Table 1:** Demographic characteristics of invited, non-respondent and respondent samples

	Invited Sample (*n* = 384)	Non-respondent sample (*n* = 275)	Respondent Sample (*n* = 109)
Age range	33 – 91 years	33–91 years	Min 38 – 84 years
Mean age (SD)	63 years (SD 11.9)	63 year (SD 11.1)	64 years (SD 10.3)
Sex: Male	*n* = 177 (46 %)	*n* = 127 (46.2)	*n* = 50 (45.9 %)
Female	*n* = 207 (54 %)	*n* = 148 (53.8)	*n* = 59 (54.1 %)
Deprivation rank^a^:			
Quartile 1 (most deprived)	157 (40.9 %)	120 (43.6 %)	37 (34.0 %)
Quartile 2	54 (14.1 %)	37 (13.5 %)	17 (15.6 %)
Quartile 3	77 (20.2 %)	57 (20.7 %)	20 (18.4 %)
Quartile 4 (least deprived)	96 (25.0 %)	61 (22.2 %)	35 (32.1 %)
High blood pressure	Not known	Not known	76 (69.7 %)
Angina	Not known	Not known	22 (20.2 %)
Myocardial infarction	Not known	Not known	12 (11.0 %)
Stroke	Not known	Not known	8 (7.3 %)
Diabetes mellitus	Not known	Not known	17 (15.6 %)
No cardiovascular morbidity	Not known	Not known	22 (20.2 %)
Current smoker	Not known	Not known	24 (22.0 %)
Ex-smoker	Not known	Not known	29 (26.6 %)

^a^Deprivation rank (grouped in quartiles) based on Northern Ireland Multiple Deprivation Index, linked to address postcode (Northern Ireland Multiple Deprivation Measure 2010, Northern Ireland Statistics and Research Agency, http://www.nisra.gov.uk/deprivation/nimdm_2010)

### Dietary behaviour

Food diaries, one month after statin prescription, showed that daily mean total fat consumption was 33.95 % of total energy intake and mean saturated fat consumption was 12.85 %, which is above recommended levels [4, 5] (Table 2). Mean daily fibre intake was 13.8 g/day, which is lower than recommended (18 g/day); fruit and vegetable intake (mean 2.7 portions/day) was also low. The mean DINE total fat score was 29 but individual scores ranged widely (from 10 to 65); the mean DINE fibre score (29) reflected low intake. One month after prescription 57.8 % (63/109) of DINE total fat scores were categorised as ‘low’; 7.3 % (8/109) were in the high fibre category. At the 4 month assessment, 60.6 % (66/109) remained in the low total fat category and 10.1 % (11/109) reported high fibre consumption.

**Table 2:** Daily intake of selected nutrients and physical activity at baseline (Month 1) and Month 4 and change undertaken

	Month 1 Mean (SD)	Month 4 Mean (SD)	Change at Month 4 Mean (95 % CI)	*p* value^*^	Intracluster Correlation Coefficient^a^ (95 % CI)	Change in score or intake at Month 4^b^		
						Increased n (%)	Decreased n (%)	No change n (%)
Food diary data (*n* = 92)								
Total fat (% of total energy)	33.95 (6.2)	3.41 (6.15)	−0.54 (−1.72,0.64)	0.36	0.04 (0.00, 0.18)	37 (40.22)	37 (40.22)	18 (19.56)
Total saturated fat (% of total energy)	12.85 (3.54)	12.18 (3.82)	−0.67 (−1.44,0.10)	0.09	0.07 (0.00, 0.23)	30 (32.61)	43 (46.74)	19 (20.65)
Monounsaturated fat (% of total energy)	10.85 (2.59)	11.16 (2.58)	0.30 (−0.13,0.74)	0.17	0.02 (0.00, 0.15)	37 (40.22)	20 (21.74)	35 (38.04)
Polyunsaturated fat (% of total energy)	5.01 (1.61)	5.38 (1.68)	0.37 (−0.01,0.75)	0.06	0.03 (0.00, 0.17)	32 (34.78)	14 (15.22)	46 (50.00)
Dietary fibre (g/day)	13.82 (5.54)	14.05 (5.76)	0.23 (−0.61,1.07)	0.58	0.10 (0.00, 0.29)	35 (38.04)	36 (39.13)	21 (22.83)
Fruit and vegetable intake (portions/day)	2.74 (1.25)	2.74 (1.36)	0.00 (−0.21,0.20)	0.96	0.00 (0.00, 0.11)	15 (16.30)	14 (15.22)	63 (68.48)
DINE questionnaire data (*n* = 109)								
Total fat score	29.39 (10.30)	29.61 (10.37)	0.22 (−1.17,1.61)	0.75	0.0 (0.00, 0.10)	42 (38.53)	46 (42.20)	21 (19.27)
Unsaturated fat score	9.40 (2.30)	9.58 (0.19)	0.17 (−0.07,0.42)	0.16	0.0 (0.00, 0.10)	10 (9.17)	6 (5.50)	93 (85.32)
Dietary fibre score	28.77 (8.70)	28.95 (10.22)	0.18 (−1.17,1.54)	0.79	0.0 (0.00, 0.10)	32 (29.36)	32 (29.36)	45 (41.28)
Physical Activity (Godin) Score (*n* = 109)	7.12 (13.9)	6.19 (13.55)	−0.93 (−2.50,0.65)	0.25	0.00 (0.00, 0.10)	4 (3.67)	9 (8.26)	96 (88.07)

^*^p value for difference between baseline (Month 1) and Month 4 using paired samples *t*-test; ^a^ based on each practice being regarded as a cluster unit; the 95 % CI of the Standard Error of the ICC is shown; ^b^ Based on per 1 unit change, for example 1 g, 1 unit score, 1 portion

Comparing dietary assessment by DINE questionnaire and food diary records found weighted Kappa analysis = 0.165 (Spearman’s correlation coefficient 0.23, *p* = 0.025) for fat intake, reflecting poor agreement but, for fibre, Kappa = 0.296 (Spearman’s correlation coefficient 0.42, *p* < 0.001), indicating fair level of agreement.

At 4 months after prescription, there was no significant change in mean saturated fat, fibre or fruit and vegetable intake, using either DINE or diary data, nor in PA (Table 2). Tracking individuals for change in food diary data (≤1 g) and DINE score (≤1 portion or unit) showed some changes but not all were in the direction of recommendations. ICCs for DINE scores showed little evidence of a cluster effect at practice level: ICCs for diary measures of change in diet showed some evidence of a cluster effect but their confidence intervals indicated a lack of precision in estimates (Table 2).

### Physical activity behaviour

Mean Godin score at one month was 7, representing low PA and did not change significantly in the following 3 months; 80.7 % at one month and 83.5 % at 4 months were insufficiently active (scores <24). Tracking individuals’ Godin scores showed some changes (≤1 unit): some increased and some decreased their activity level (Table 2).

### Recall of advice and behaviour change

Only 58 participants (50.5 %) reported having received advice about their diet or PA. Of these, 38 (35 %) recalled having received it from their GP or practice nurse; 20 (18 %) recalled advice from a hospital doctor, nurse or dietician. No significant cluster effect was found for practices. Overall, 4 people reported having received lifestyle advice from sources other than the GP surgery or hospital: these included online information, family, a commercial weight loss programme and a community dietician (See Additional file 1).

Relatively more participants reported having made positive dietary changes at the time of their first statin prescription than in the following 3 months (37 (33.9 %) v 14 (12.8 %)). Only 3 reported having increased their PA at either time. Among those who recalled having received advice, 69.0 % (40/58) reported having changed their diet or PA, compared to 31.4 % (16/51) of those who did not recall being given advice.

There was consistency in respect of adherence to healthy lifestyle advice: those who were physically active (Godin score ≥24), had lower levels of fat and saturated fat intake and higher levels of fibre, fruit and vegetable intake than those who were less active (Table 3).

**Table 3:** Nutrient intake^a^ of inactive and physically active participants at Month 1

	Physically inactive^b^	Physically active^c^	p value^*^
	(*n* = 75)	(*n* = 17)	
Total fat (% of total energy)	34.75 (5.80)	30.42 (6.83)	<0.01
Saturated fat (% of total energy)	13.43 (3.36)	10.31 (3.25)	<0.01
Monounsaturated fat (% of total energy)	11.08 (2.44)	9.88 (3.06)	0.15
Polyunsaturated fat (% of total energy)	5.04 (1.62)	4.88 (1.59)	0.72
Dietary fibre (gm/day)	13.25 (4.59)	16.30 (8.31)	0.04
Fruit and vegetable intake (portions/day)	2.60 (1.16)	3.37 (1.49)	0.02

^a^Nutrient intake based on 4 day food diary;^b^Godin physical activity score <24;^c^Godin physical activity score ≥24

^*^p value for significant difference between groups (independent samples *t*-test)

## Discussion

Our study findings show that initiation of statin prescription in a UK population was not associated with adoption of healthy diet or physical activity behaviours for a majority of patients. Whilst approximately one third of our sample reported having improved their diet around the time of their first statin prescription, questionnaire data at 1 month and 4 months later show that approximately 40 % continued to consume higher levels of fat than advised, 55 % consumed low levels of fibre and over 80 % were insufficiently active for health benefits. Only half of our sample recalled having received lifestyle advice from a health professional. Those who recalled having received lifestyle advice were more likely, than those who did not, to report associated behaviour change.

### Comparison with previous literature

To our knowledge, this is the first study to report diet and physical activity behaviours following initiation of statin therapy for patients in Europe. Whilst ethics approval did not allow us to confirm cardiovascular risk or morbidities by accessing medical records, self-reported cardiovascular morbidity indicated that half of our sample were prescribed statins for secondary prevention. In keeping with a recent survey of 24 centres providing secondary prevention across Europe [14], our findings show a need for improved diet and physical activity behaviour to optimise reduction of cardiovascular risk.

Healthy lifestyles are not an alternative strategy to statin prescription; both are important ways of reducing cardiovascular risk and recent studies suggest that the effects of taking a statin and increasing exercise are additive [15]. Adherence to lifestyle advice tends to fall over time [17, 18] so it may be expected that our patients’ lifestyles are unlikely to improve after a longer time interval than we observed. Recent guidance, extending statin prescribing for primary prevention of cardiovascular disease to individuals with a 10 % 10-year risk [5] will result in a substantial additional number of people in the UK receiving statin therapy and requiring advice and support for lifestyle behaviour change: its implications for general practice services and for patients should be fully recognised. This has particular significance in the context of this study which was set in Northern Ireland, an area of high cardiovascular disease prevalence.

Behaviour patterns can be strongly influenced by people’s social, cultural or physical environmental circumstances and there are recognised gaps in our understanding of how people’s knowledge, attitudes and behaviour are linked [25]. However, lifestyle interventions in primary care can be effective for behaviour change [26] and knowledge exchange between the patient and health professional is an important step within these. Recent research has reported that dietary counselling by primary care clinicians does not typically contain consistent, clear suggestions for specific change in behaviours [27]. Lack of clear advice may have contributed to our finding that some participants reported they did not receive any advice, yet they reported having made changes to their behaviour. Experiential learning of different approaches to promoting physical activity may help healthcare practitioners to plan and deliver appropriate advice in their future practice [28]. A similar educational approach may be relevant to the promotion of healthy diets.

### Strengths and limitations of the study

Our data were gathered in 2005/06 but, whilst there has been increasing emphasis on statin prescribing, with statin consumption per capita in the UK being the highest of all European countries, [3] there is little evidence of a concomitant focus on improving the diet and PA behaviours of patients taking statins and little is known of patients’ diet and PA habits following initiation of statin prescription. Thus we consider that, whilst the study has limitations in its methodology, our findings have contemporary relevance.

We achieved a high level of retention of participants within the study and of satisfactory completion of outcome measures. The DINE and Godin questionnaires were chosen because they were validated, short and easy to administer. We did not meet face-to-face with participants as we wished to minimise the impact of our contact on their behaviour but our decision to administer the questionnaires by telephone avoided incomplete or invalid completion of responses, which has been reported previously with postal administration. [29] Telephone administration was efficient and appeared acceptable to participants, with 89.3 % completing second questionnaires.

Our novel approach, with concurrent administration of a telephone questionnaire and completion of a 4-day food diary, allowed direct comparison of these outcome measures in a primary care setting. Their level of correlation in diet fibre assessment indicates that the questionnaire, which is readily applicable to practice, gives relevant information to a clinician. The poorer correlation between questionnaire and diary record in assessing fat consumption corresponds to previous reports of under-reporting of total energy and fat intake compared to objective measurements [30]. Self-report may also over-estimate PA, so that true levels of PA may be underestimated within the sample [31].

Our study is limited in data about non-participants, to inform the extent of respondent bias. Ethics approval did not allow reminders or alternative approaches to invite participation: a reminder letter or telephone contact from the patient’s primary care team may have increased our rate of recruitment [32]. Provision of resources to support greater involvement in the study’s administration by practice staff, allowing replies to be sent to the practice rather than the researcher, personal invitations from clinicians to patients, or information about the study being displayed in the practice premises may also have boosted recruitment. Nevertheless, our sample of responsive patients who had recently begun taking statins has shown that management of cardiovascular risk reduction is sub-optimal. Our respondents included a relative, though statistically non-significant, over-representation of people from more affluent areas who are more likely to lead healthier lifestyles than those in deprived areas. Also, our respondents may well be those who were more interested in and aware of lifestyle factors and not entirely representative of all those who are prescribed statins, so that our non-responders may have even greater need for lifestyle change.

We acknowledge that we did not measure diet and PA at the point of prescription but we did not intervene in consultations because we wished to examine real-world behaviour and outcomes from usual care, without other influences. Whilst we did not include a control group of patients who were not prescribed statins, to determine if their behaviours differed from our sample, another recent study indicates that statin users’ and non-users’ behaviours are similar [16]. In order to minimise the potential impact of our contact on participants’ behaviour we limited the detail of questions regarding recall of advice, so that we did not collect data regarding the content or format of advice received. Also, we did not have access to medical records to confirm whether or not patients received lifestyle advice but we interpret failure to recall advice as meaning that it was either not given or not perceived as being helpful. Whilst we could not confirm the proportion of our sample who started statins for primary prevention, 20 % had started therapy with no reported cardiovascular disease: it would be of interest to determine the level of risk at which this had been prescribed. Our finding that half of the participants reported that they did not recall having been given advice, yet one third of these reported having changed their diet or PA habits, would justify further study of how lifestyle advice is disseminated, how it is provided for patients in everyday practice, and of its effectiveness.

### Conclusions and implications for practice

Our findings show that diet and PA lifestyles remain essentially unchanged for many patients after starting a statin, with higher consumption of saturated fat, less dietary fibre and lower levels of physical activity than recommended. Since only about half of our sample reported that they recalled having received lifestyle advice, there would appear to be a need for a greater emphasis on the delivery of this in practice within consultations. How this may be achieved with current workload pressures in general practice requires further study.

General practitioners who understand their patients’ social and physical environments and their capability to increase their physical activity or change their diet are ideally placed to help them. Involving families with patients in preventive programmes promotes the successful adoption of healthy lifestyles [33]. Further research is needed to develop strategies for efficient collaborative working between general practice and other agencies, so that advice given to individuals by practitioners is set in the context of community support for health promoting behaviours.

## Abbreviations

DINE, dietary intervention in primary care; GP, general practitioner; ICCs, intracluster correlation coefficients; PA, physical activity

## Additional file

Additional file 1: Online SPSS STATISTICS. (SAV 85 kb)

## References

[1] Abramson JD, Rosenberg HG, Jewell N, Wright JM (2013). Should people at low risk of cardiovascular disease take a statin?. BMJ.

[2] Borland S. Statin prescriptions treble in ten years to hit almost 60 million: NHS spends £100m handing out pills to seven million Britons. The Mail Online. 2014, 9 July. http://www.dailymail.co.uk/health/article-2686744/Statin-prescriptions-treble-ten-years-hit-60million-NHS-spends-100m-handing-pills-seven-million-Britons.html. Accessed 19 Oct 2015.

[3] OECD. Health at a glance 2013. The Organisation for Economic Co-operation and Development. www.oecd.org/els/health-systems/health-at-a-glance.htm. Accessed 8 Aug 2014.

[4] Stone NJ, Robinson J, Lichtenstein AH (2014). 2013 ACC/AHA Guideline on the Treatment of Blood Cholesterol to Reduce Atherosclerotic Cardiovascular Risk in Adults: A Report of the American College of Cardiology/American Heart Association Task Force on Practice Guidelines. Circulation.

[5] National Institute for Health and Care Excellence. Cardiovascular disease: risk assessment and reduction, including lipid modification. https://www.org.uk/guidance/cg181. Accessed 10 July 2016.

[6] Perk J, De Backer G, Gohlke H, Graham I, Reiner Z, Verschuren WMM (2012). European Guidelines on cardiovascular disease prevention in clinical practice (version 2012). The Fifth Joint Task Force of the European Society of Cardiology and Other Societies on Cardiovascular Disease Prevention in Clinical Practice. Eur Heart J.

[7] Malhotra A (2013). Saturated fat is not the major issue. BMJ.

[8] Slavin JL, Llyod B (2012). Health Benefits of Fruits and Vegetables. Adv Nutr.

[9] Sofi F, Cesari F, Abbate R, Gensini GF, Casini A (2008). Adherence to Mediterranean diet and health status: meta-analysis. BMJ.

[10] Bull FC, and the Expert Working Groups. Physical Activity Guidelines in the UK: Review and Recommendations. School of Sport, Exercise and Health Sciences, Loughborough University, May 2010. https://www.gov.uk/government/uploads/system/uploads/attachment_data/file/213743/dh_128255.pdf. Accessed 11 July 2016.

[11] US Department of Health and Human Services. 2008 Physical Activity Guidelines for Americans. 2008. http://www.health.gov/paguidelines/pdf/paguide.pdf. Accessed 11 July 2016.

[12] Unal B, Critchley JA, Capewell S (2004). Explaining the decline in coronary heart disease mortality in England and Wales between 1981 and 2000. Circulation.

[13] Hughes J, Kee F, O’Flaherty M (2013). Modelling coronary heart disease mortality in Northern Ireland between 1987 and 2007: broader lessons for prevention. Eur J Prev Cardiol.

[14] Kotseva K, Wood D, De Bacquer D et al., on behalf of the EUROASPIRE Investigators. EUROASPIRE IV: A European Society of Cardiology survey on the lifestyle, risk factor and therapeutic management of coronary patients from 24 European countries. European J Prev Cardiol. 2015, doi: 10.1177/2047487315569401 10.1177/2047487315569401 25687109

[15] Kokkinos PF, Faselis C, Myers J, Panagiotakos D, Doumas M (2013). Interactive effects of fitness and statin treatment on mortality risk in veterans with dyslipidaemia: A cohort study. Lancet.

[16] Savolainen J, Kautiainen H, Niskanen L, Mäntyselkä P (2015). Decreasing cholesterol levels in the community – lifestyle change with statin?. BMC Fam Pract.

[17] Cupples ME, McKnight A (1999). Five year follow up of patients at high cardiovascular risk who took part in randomised controlled trial of health promotion. BMJ.

[18] Chow CK, Jolly S, Rao-Melacini P, Fox KA, Anand SS, Yusuf S (2010). Association of diet, exercise, and smoking modification with risk of early cardiovascular events after acute coronary syndromes. Circulation.

[19] Mann DM, Allegrante JP, Natarajan S (2007). Dietary indiscretion and statin use. Mayo Clin Proc.

[20] Roe L, Strong C, Whiteside C, Neil A, Mant D (1994). Dietary intervention in primary care: validity of the DINE method for diet assessment. Fam Pract.

[21] Godin G, Shephard RJ (1985). A simple method to assess exercise behavior in the community. Can J Appl Sport Sci.

[22] Food standards agency. National Diet & Nutrition Survey: Adults aged 19 to 64. Volume 5 2004. tna.europarchive.org/20110116113217/http:/www.food.gov.uk/multimedia/pdfs/ndnsfour.pdf. Accessed 11 July 2016.

[23] Crawley H. Food portion sizes. Fish & Food, Min. of Agriculture. (Paperback) Eds. Mills A, Patel S. The Stationery Office Books, London. 1994, 1 February. ISBN-13 9780112429616

[24] Department of Health. Dietary Reference Values for Food Energy and Nutrients for the United Kingdom. Report of the Panel on Dietary Reference Values of the Committee on Medical Aspects of Food Policy. Report on Health and Social Subjects no. 41. London: HSMO; 1991 1961974

[25] NICE guidelines [PH49]. Behaviour change: individual approaches. National Institute for Health and Care Excellence. January 2014 https://www.nice.org.uk/Guidance/PH49 Accessed 20 Oct 2015.

[26] Cole JA, Smith SM, Hart N, Cupples ME (2011). Systematic Review of the Effect of Diet and Exercise Lifestyle Interventions in the Secondary Prevention of Coronary Heart Disease. Cardiol Res Pract.

[27] Phillips K, Wood F, Spanou C, on behalf of the PRE-EMPT Team (2012). Counselling patients about behaviour change: the challenge of talking about diet. Br J Gen Pract.

[28] Cooke PA, Tully MA, Cupples ME, Gilliland AE, Gormley GJA (2013). Randomised Control Trial of Experiential Learning to Promote Physical Activity. Educ for Prim Care.

[29] Cole JA, Gillespie P, Smith SM, Byrne M, Murphy AW, Cupples ME (2014). Using postal questionnaires to evaluate physical activity and diet behaviour change: case study exploring implications of valid responder characteristics in interpreting intervention outcomes. BMC Res Notes.

[30] Macdiarmid J, Blundell J (1998). Assessing dietary intake: Who, what and why of under-reporting. Nutr Res Rev.

[31] Prince SA, Adamo KB, Hamel ME, Hardt J, Connor Gorber S, Tremblay M (2008). A comparison of direct versus self-report measures for assessing physical activity in adults: a systematic review. Internat J Behav Nutr Phys Act.

[32] Leathem CS, Cupples ME, Byrne MC, O’Malley M, Houlihan A, Murphy AW, Smith SM (2009). Identifying strategies to maximise recruitment and retention of practices and patients in a multicentre randomised controlled trial of an intervention to optimise secondary prevention for coronary heart disease in primary care. BMC Med Res Methodol.

[33] Wood DA, Kotseva K, Connolly S (2008). Nurse-coordinated multidisciplinary, family-based cardiovascular disease prevention programme (EUROACTION) for patients with coronary heart disease and asymptomatic individuals at high risk of cardiovascular disease: a paired, cluster-randomised controlled trial. Lancet.

